# Lactate clearance as a predictor of mortality in colonic perforation

**DOI:** 10.1186/cc13365

**Published:** 2014-03-17

**Authors:** R Egashira, T Kobayashi

**Affiliations:** 1 Osaki Citizen Hospital, Miyagi, Japan

## Introduction

The objective of this study was to determine whether lactate clearance (LC) is a significant indicator of mortality in patients with colorectal perforation. LC has been associated with mortality in heterogeneous critically ill patients, but its role as a predictor of mortality in homogeneous patients with colorectal perforation is unclear.

## Methods

We retrospectively analyzed the clinical data of patients who underwent emergency surgery for colorectal perforation and were admitted to the ICU of our hospital from January 2003 to August 2013. Patients with traumatic, iatrogenic, and appendicitis perforations were excluded. The primary endpoint was survival to hospital discharge. The modified Sequential Organ Failure Assessment (mSOFA) score, a customized SOFA score excluding the central nervous system component [1], was used for prognostic scoring. The mSOFA score and several clinical factors were analyzed by univariate analysis as possible predictors of survival. We collected lactate levels and base excess (BE) measured during surgery and at 6, 12, and 24 hours after the first measurement and calculated the respective LC values. The associations of initial blood lactate level, LC, and Be with mortality were assessed by receiver operating characteristics (ROC) curve and logistic regression analyses.

## Results

Of the 61 patients identified, five were excluded as their ICU stay was <24 hours. The overall mortality in the remaining 56 patients (mean age of 76.7 ± 10.4 (SD) years) was 21.4%. In univariate analysis, mSOFA and several other variables correlated significantly (*P *< 0.05) with mortality. The area under the ROC curve for LC at 6, 12, and 24 hours was 0.601, 0.719, and 0.731, respectively. LC at 24 hours was the most accurate, and its optimum cutoff value was 37.5%. LC, lactate level, and BE at 24 hours, as well as the significant factors in univariate analysis, were entered into a stepwise logistic regression model, which revealed 24-hour LC ≤37.5% (odds ratio (OR), 23.0) and mSOFA score (OR, 2.1) as independent predictive values of mortality.

## Conclusion

In patients with colorectal perforation, 24-hour LC is more accurate than LC measured at earlier time points. Patients with 24- hour LC ≤37.5% and a high mSOFA score have a high risk of in-hospital mortality.
